# Anaesthetic efficacy of intraligamentary injection compared to incisive nerve block using 3% mepivacaine hydrochloride: a randomized clinical trial

**DOI:** 10.1186/s12903-024-05147-z

**Published:** 2025-01-17

**Authors:** Suzan Salem, Islam Saad, Ramy Elmoazen, Ghada Amin Khalifa

**Affiliations:** 1 Department of Oral and Maxillofacial Surgery, Alexandria University Hospitals, Alexandria University, Alexandria, Egypt; 2 Department of Oral and Maxillofacial Surgery, Faculty of Dentistry, Alexandria University, Alexandria, Egypt; 3 Department of Oral Medicine, Periodontology, Oral diagnosis and Radiology, Alexandria University Hospitals, Alexandria University, Alexandria, Egypt; 4 Department of Oral medicine, Periodontology, Diagnosis & Oral Radiology, Faculty of Dentistry, Alamein International University, Alamein, Egypt; 5 School of Computing, University of Eastern Finland, Yliopistokatu 2, Joensuu, 80100 Finland; 6 Maxillofacial Surgery and Diagnostic Science, College of Dentistry, Qassim University, Buraydah, Saudi Arabia; 7 Faculty of Dental Medicine for Girls, Al Azhar University, Cairo, Egypt

**Keywords:** Anxiety, Incisive nerve block, Intraligamentary injection technique

## Abstract

**Background:**

In dentistry, local anesthetic is frequently used to manage pain throughout several phases of dental treatments, including tooth extraction. The study aimed to compare the effectiveness of two techniques for controlling pain during mandibular exodontia (tooth extraction), specifically focusing on the pain experienced during injection and extraction of mandibular anterior and premolars teeth. The two techniques being compared are the intraligamentary injection technique (ILI) and the incisive nerve block technique (INB).

**Materials and methods:**

In this study, 100 mandibular anterior and premolars and teeth that were indicated for extraction were included. The effectiveness of the two local anaesthesia techniques, intraligamentary injection technique (ILI) and incisive nerve block (INB), were compared using Modified Dental Anxiety Scale for Dental Extraction Procedure (MDAS-DEP) and visual analogue scale (VAS) during the injection and extraction stages of the procedure.

**Results:**

A total of 100 participants (42 females, 58 males) with a mean age of 50.97 ± 11.59 years took part in the study. The mean VAS score in the INB group was 6.14 after injection and 3.86 after extraction, while in the ILI group, it was 5.46 and 2.90, respectively. There was a statistically significant difference between the two groups both after injection (*p* = 0.001) and extraction (*p* < 0.001), as well as within each group (Control: *p* < 0.001; Study: *p* < 0.001). For MDAS-DEP, the INB group had mean scores of 15.86 and 11.26 after injection and extraction, respectively, while the ILI group had scores of 15.68 and 10.94, showing a significant difference within each group after both injection (*p* < 0.001) and extraction (*p* = 0.001). However, no significant difference was found when comparing MDAS-DEP scores between the two groups from injection to extraction (*p* = 0.802).

**Conclusion:**

The intraligamentary injection technique (ILI) appears less painful during injection and provides profound pain relief during extraction. The results suggest that ILI can be used as a sole anaesthetic technique during extraction of lower anterior and premolar teeth.

**Trial registration:**

This trial was retrospectively registered on 27/01/2023 with the identifier ISRCTN83272316 in Isrctn.com.

**Supplementary Information:**

The online version contains supplementary material available at 10.1186/s12903-024-05147-z.

## Background

Providing effective pain control is a crucial aspect of dental care. Pain management during dental procedures is important to ensure patient comfort, reduce anxiety, and improve the overall dental experience. Adequate pain control can also help to prevent complications and ensure a successful outcome for the procedure and this can be achieved by using a potent anaesthetic drugs and an less discomfort anaesthetic technique [1]. 

Several anaesthetic drugs are used for years for dental procedures. Mepivacaine was introduced into dentistry in 1960 and is the third most widely used local anaesthetic solution in dentistry after articaine and lidocaine [2–4]. It has the same potency as lidocaine but is milder in vasodilation, leading to a longer duration of anaesthesia. Mepivacaine is available as a 2% or 3% solution with or without vasoconstrictors. Several studies recommend using 3% mepivacaine as it has quick onset, a greater absorption of the anaesthetic in the maxillary anterior region, ideal anaesthetic effect and little side effect on cardiovascular system [5–8]. 

Moreover, different anaesthetic techniques, such as infiltration and nerve block, are used during dental procedures depending on the location of the tooth treated. However, these techniques can be associated with complications, such as pain, nerve injury, and trismus [9]. Yes, fear of injections is a common phobia known as needle phobia or trypanophobia, which can result in anxiety or avoidance of medical procedures that require injections, including dental or medical procedures that use local anaesthesia [10]. To help mitigate this fear, healthcare providers may employ techniques to reduce discomfort during injections, improving the patient experience and satisfaction [11–13]. 

Although the inferior alveolar nerve block (IANB) is frequently used to extract mandibular teeth, but it can have associated complications such as pain, difficulty opening the mouth (trismus) and in rare cases nerve damage and temporal facial nerve paralysis [14]. Another challenge is the inconsistent availability of reliable landmarks for the IANB technique, which can lead to difficulty in accurately administering the injection [15]. 

In order to address the problems connected with traditional techniques like the inferior alveolar nerve block (IANB), new anaesthetic techniques like the intraligamentary injection technique (ILI) and incisive nerve block (INB) have been developed. The goal of these alternative methods is to reduce the incidence of pain, nerve damage, and other adverse effects, and improve patient comfort and satisfaction during dental procedures [9, 15]. 

The incisive nerve block (INB) is a commonly used alternative to the inferior alveolar nerve block (IANB) for anesthetizing mandibular anterior and premolars teeth during dental procedures. Studies have shown that the INB provides an additional benefit of local infiltration along with the regional block of the incisive nerve, leading to a high success rate for anesthetizing mandibular anterior and premolars teeth [16]. 

On the other hand, intraligamentary anaesthesia which was first developed by Chompret [17] offers advantages over inferior alveolar nerve block (IANB) in situations when there are anatomical variances, also beneficial in cases where a limited area needs to be anesthetized, such as in a single-tooth extraction in a quadrant, or to avoid bilateral IANB and in treatment of children. Additionally, ILI can be a useful tool in the diagnosis of mandibular pain [18, 19]. Moreover, it was suggested as a sole anaesthetic technique in various dental procedures in place of inferior alveolar nerve block (IANB) [20–22]. 

Up to our knowledge, the anaesthetic effectiveness of ILI and INB employing 3% mepivacaine hydrochloride in the extraction of mandibular anterior/premolar teeth has not been studied. This study’s main objective was to compare the anaesthesia success rates of ILI and INB using 3% mepivacaine hydrochloride on mandibular anterior and premolar teeth that were going to be extracted. The secondary goal is to ascertain whether method for anaesthetizing and extracting mandibular anterior/premolar teeth is more tolerated by patients.

## Method

### Study design and setting

The study was a randomized, single-blind, controlled clinical trial conducted at the outpatient clinic of the College of Dentistry at Qassim University in Saudi Arabia from January 2019 to June 2019. Participants were divided into two groups with a 1:1 ratio, Group A receiving INB using 3% mepivacaine hydrochloride and the group B receiving ILI using the same anaesthetic. The study aimed to compare and determine the superiority of the two techniques.

### Ethical consideration

The study was approved by the Dental Ethical Committee of College of Dentistry, Qassim University with the code EA/6015/2018 and was retrospectively registered on 27/01/2023 with the identifier ISRCTN83272316 in Isrctn.com. The study was conducted following the guidelines of the Helsinki Declaration and its amendments. Reporting of the study follows the protocol established by the Consolidated Standards of Reporting Trials Statement (CONSORT) checklist.

### Sample size determination

The sample size was calculated based on results from previous studies and considering a study Power 1 − β1 of 0.8 and Type I error rate, α 5%. The sample size was calculated on comparing means of previous study and was estimate to be 50 per group and total size to be 100.

### Randomization and participants allocation

In this clinical trial, the allocation of participants was performed using two sealed envelopes containing the two intervention techniques. A dental auxiliary delivered the envelopes to the participants and asked them to choose one, ensuring equal distribution of potential confounding factors. Both the operator and the participant were blinded to the intervention technique when selection, resulting in a single-blind study design.

### Eligibility criteria

The study included healthy patients of both genders, between the ages of 25 and 70 years old, who had non-restorable mandibular anterior and premolar teeth and were willing to participate after fully understanding oral instructions. Patients with certain medical conditions (malignant neoplasm, cardiac pacemaker, diabetes, epilepsy), pregnant women, and those with periodontally compromised teeth or acute dentoalveolar infection and those required osteotomy for extraction were excluded from the study.

### Intervention

All participants signed an informed consent form. Before injection, Modified Dental Anxiety Scale for Dental Extraction Procedure (MDAS-DEP) [23](Appendix 1) The MDAS-DEP consists of five questions, which are **(1)** If you were told that one of your teeth had to be extracted, how would you feel; **(2)** If you were about to go to the dentist tomorrow to have your tooth extracted, how would you feel; **(3)** If you were sitting in the waiting room waiting for your dental extraction procedure, how would you feel; **(4)** If you were about to get a local anaesthesia injection in your gum, how would you feel; and **(5)** If your third molar was about to be removed through a surgical procedure, how would you feel. As for the answer, the five Likert-scale answers used in the MDAS-DEP are the same answers used in the original MDAS [23, 24].

**Fig. 1:**
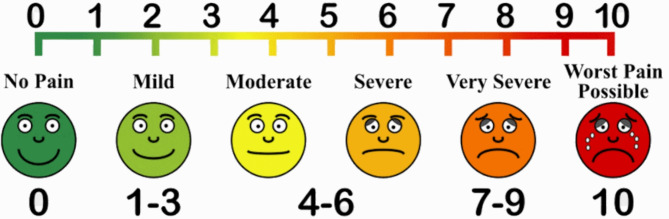
Visual Analogue Scale (VAS)

Also, participants were instructed on how to use the Visual Analog Scale (VAS) to rate their pain before any injection. The VAS was 10 cm long and divided into six categories with faces expression, with no pain scale 0, mild pain scale 1–3, moderate pain scale 4–5, severe pain scale 5–6, very sever 7–9 and worst pain possible scale 10 (Fig. 1).

A topical anaesthetic was applied at the injection site ad a standard 27-gauge short needle loaded with 3% mepivacaine hydrochloride were used for both techniques.

For group A, the needle was directed towards the mucobuccal fold just anterior to the mental foramen which is typically located between the apices of the two mandibular premolars [12] with a depth of penetration of 5 to 6 mm. A dose of 0.6 mL of LA (about one-third of a cartridge) was given over 30 s, followed by 0.3 mL of LA solution for the lingual side.

On the other hand, in group B the needle was inserted parallel to the tooth’s long axis and up to the depth of the gingival sulcus on four sites “mesio- and disto-lingual, and mesio- and disto-buccal” [22]. On the mesial line angle of the tooth to be anesthetized the needle inserted as far apically as possible. A dose of 0.2 mL of local anaesthetic (LA) was administered over 20–30 s. Then the same technique is performed on the remaining three sites.

Following administration of anaesthetic injections, patients completed the scales to assess the amount of pain they experienced for each injection using MDAS-DEP and VAS.

Successful anaesthesia was determined by testing the patient’s numbness using a dental probe. The probe was placed on the gingiva immediately after the injection and at intervals of 5–10 s until the patient was fully numb. Extraction was performed following the best practice guidelines and the participants were request to evaluate the extraction pain using the same scales.

### Validity and reliability

All clinical procedures were performed by one trained and calibrated operator to reduce the bias. Another operator was responsible for collecting pain scales. Intra-examiner reliability was tested by Intraclass correlation (ICC) [25]. 

### Data analysis

All statistical analysis was performed with Statistical Package for Social Sciences (SPSS) software version 27. Independent Student’s t-test was used to compare the parametric data (i.e., Mean Modified Dental Anxiety Scale, mean injection pain, and mean extraction pain). Repeated measures ANOVA was used to compare mean post-injection pain. The success rate was calculated as the percentage of patients with zero or mild pain during the procedure. The results were considered statistically significant if the probability (p-value) of obtaining the results by chance is less than 0.05.

## Results

One hundred twenty-five patients were assessed for eligibility. Thirteen patients were excluded due to systemic diseases and periodontal compromised condition of the teeth and twelve patients refuse to participate for personal reasons. The remaining 100 participants, 58 male and 42 females, with a mean age of 50.97 ± 11.59, were randomly allocated into two study groups, with 50 in the control group (Group A) and 50 in the study group (Group B) (Table 1).

**Table 1:** Distribution of participants regarding among the study groups

Variable	INB (Control group)	INI (test group)	Total	*P* value
Age (Mean ± SD)	51.16 ± 11.09	50.88 ± 11.95	50.97 ± 11.59	0.31
N (%)				
Female Male	18 (36) 32 (64)	24 (48) 26 (52)	42 (42) 58 (58)	0.77
Tooth location N (%)				
Left Central Incisor Left Lateral Incisor Left Canine Left First Premolar Left second Premolar Right Central Incisor Right Lateral Incisor Right Canine Right First Premolar Right second Premolar	5.0 5.0 2.0 5.0 5.0 8.0 4.0 5.0 6.0 5.0	5.0 4.0 4.0 4.0 5.0 7.0 3.0 5.0 8.0 5.0	10.0 9.0 6.0 9.0 10.0 15.0 7.0 10.0 14.0 10.0	0.87

**Fig. 2:**
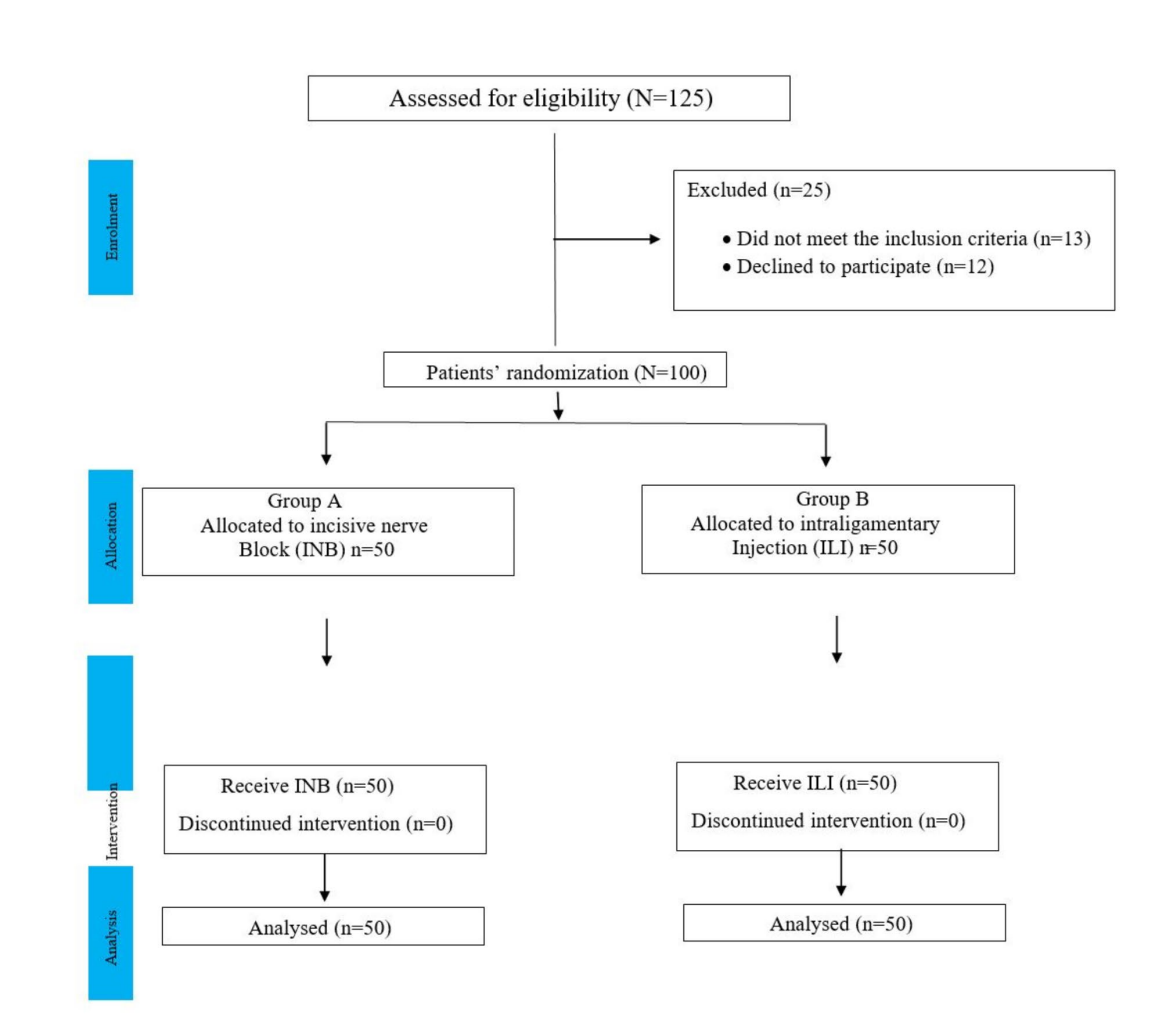
Consort flow chart

The most extracted tooth was the mandibular right central incisor, while the least extracted tooth was the mandibular left canine. There were no significant differences between the control and study groups in terms of age, gender, or tooth location. (*p* = 0.31, 0.77, 0.87) respectively. The study did not encounter any failures with INB, so no further injections were needed. The participant’s eligibility, allocation, intervention, and data analysis are illustrated in consort flow chart (Fig. 2).

**Fig. 3:**
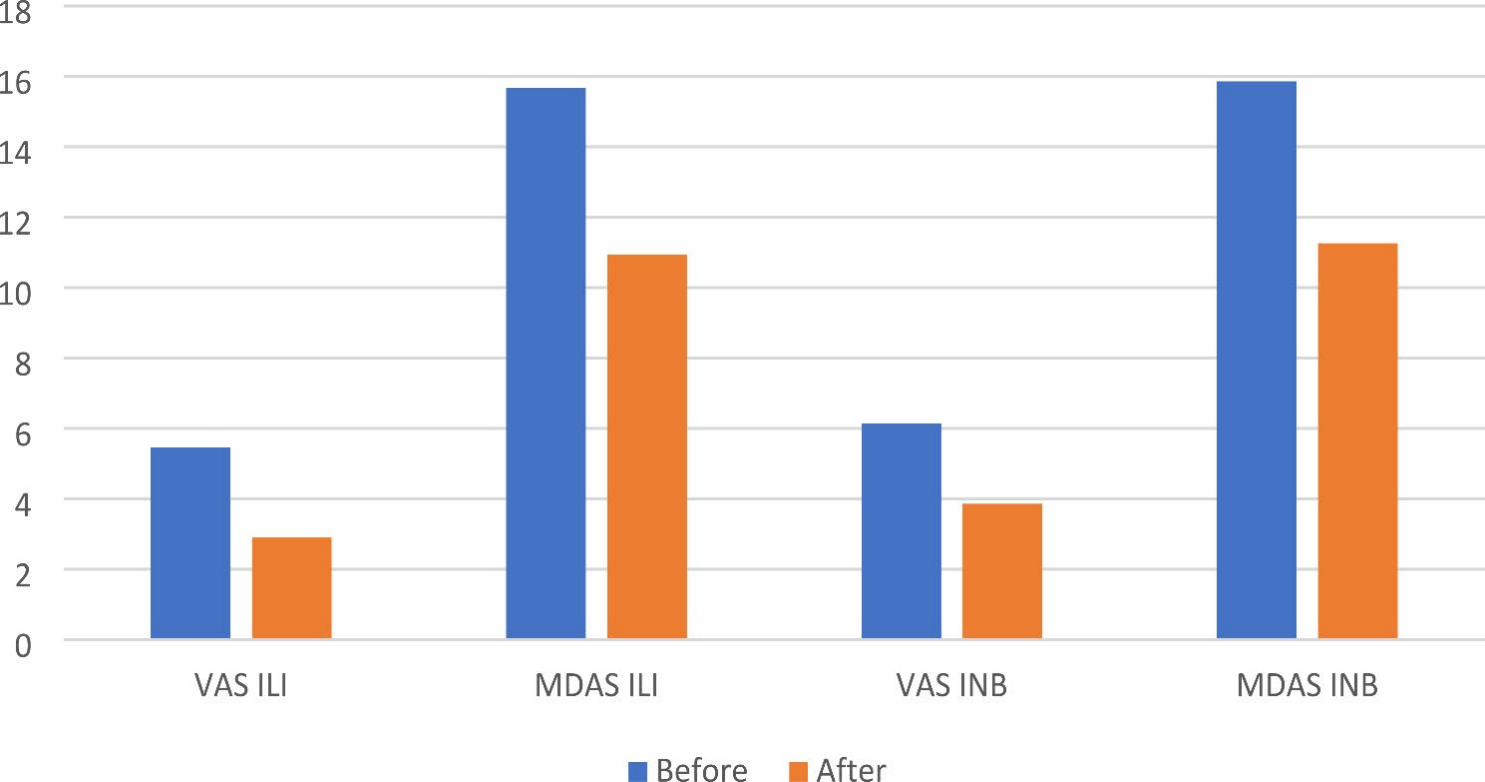
Comparison between VAS scores and MDAS-DEP for the control group and the study group before and after extraction

**Fig. 4:**
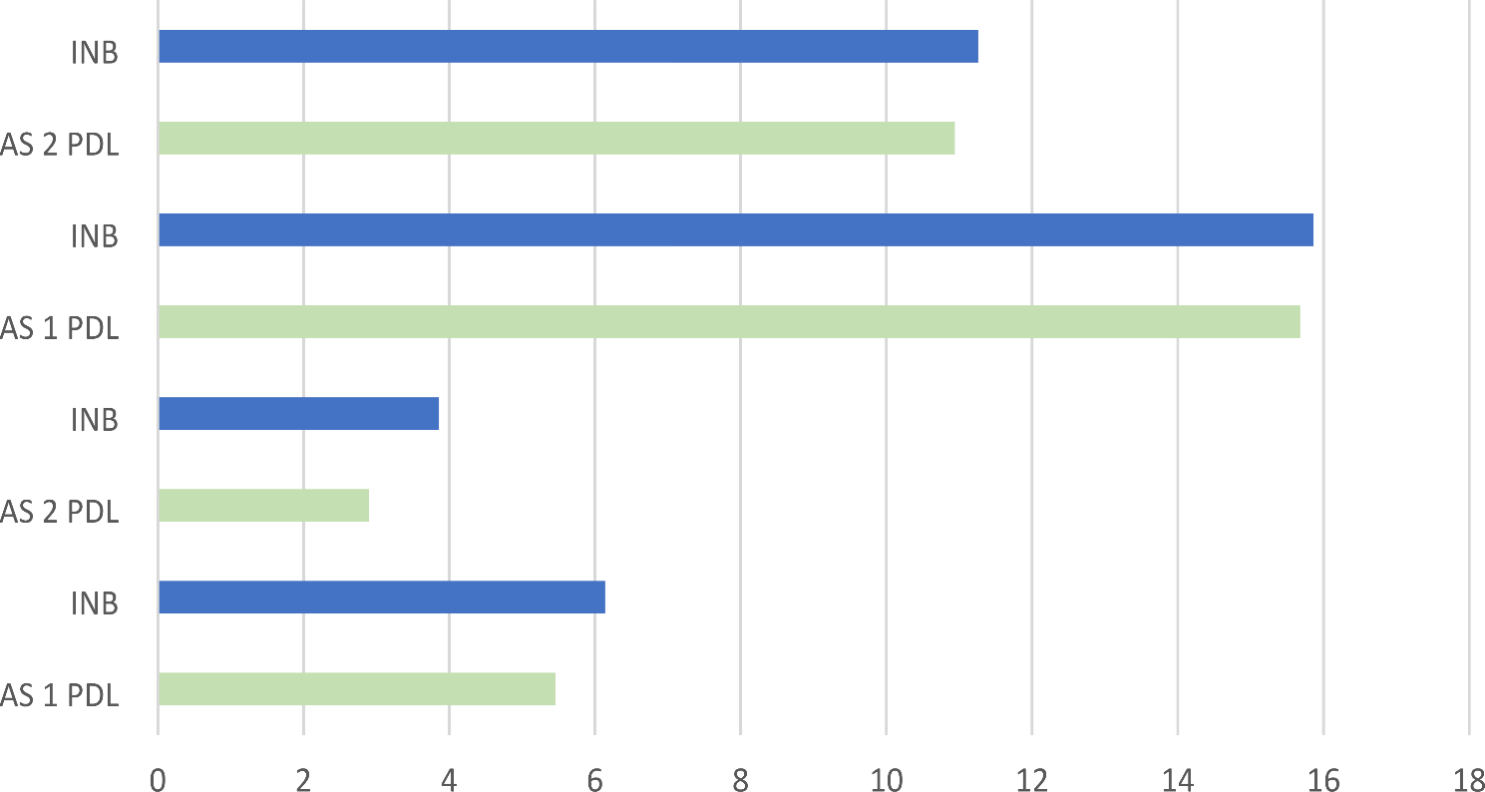
Comparison between VAS scores and MDAS-DEP between the control group and the study group

Upon analysing pain using the VAS, the mean VAS score in the INB group was 6.14 after injection and 3.86 after extraction, while in the ILI group, it was 5.46 and 2.90, respectively, a statistically significant difference was observed between the two study groups in pain levels after injection (*p* = 0.001) and extraction (*p* < 0.001) (Table 2). Additionally, there was a significant difference in VAS scores when comparing injection and extraction within each group (Control: *p* < 0.001, Study: *p* < 0.001) (Table 3) (Figs. 3 and 4).

**Table 2:** Comparison between VAS scores and MDAS-DEP for the control group and the study group before and after extraction

Before – After			95% Confidence Interval of the Difference		Paired t-test	*p*
	Mean	Std. Deviation	Lower	Upper		
VAS1 ILI	5.4600	0.95212	2.31532	2.80468	21.026	*P* < 0.001*
VAS 2 ILI	2.9000	0.76265				
MDAS 1 ILI	15.6800	3.96639	4.00561	4.83439	21.434	*P* < 0.001*
MDAS 2 ILI	10.9400	3.33448				
VAS 1 INB	6.1400	0.96911	2.08923	2.47077	24.017	*P* < 0.001*
VAS 2 INB	3.8600	0.90373				
MDAS 1 INB	15.8600	3.15588	4.45411	5.38589	21.222	*P* < 0.001*
MDAS 2 INB	11.2600	3.59597				

**Table 3:** Comparison between VAS scores and MDAS-DEP between the control group and the study group

2 Groups comparison				95% Confidence Interval of the Difference		t-test	*p*
		Mean	Std. Deviation	Lower	Upper		
VAS 1	PDL	5.4600	0.95212	-1.06128	-0.29872	-3.539	0.001*
	INB	6.1400	0.96911				
VAS 2	PDL	2.9000	0.76265	-1.29187	-0.62813	-5.740	*P* < 0.001*
	INB	3.8600	0.90373				
MDAS 1	PDL	15.6800	3.96639	-1.60251	1.24251	-0.251	0.802
	INB	15.8600	3.15588				
MDAS 2	PDL	10.9400	3.33448	1.05630	-1.69630	0.461	0.802
	INB	11.2600	3.59597				

Regarding the MDAS-DEP scales, the results indicated a statistically significant difference within each group after injection (*p* < 0.001) and extraction (*p* = 0.001) (Table 2). The mean MDAS-DEP score in the INB group was 15.86 ± 3.156 during injection and 11.26 ± 3.60 during extraction, slightly higher than the ILI group, which had scores of 15.68 ± 3.40 and 10.94 ± 3.16, respectively. However, there was no significant difference in comparing MDAS-DEP scores from injection to extraction between the control group and the study group (*p* = 0.802) (Table 3) (Figs. 3 and 4).

## Discussion

A previous negative dental experience, especially one involving local anaesthesia, has been associated with heightened dental fear, uncooperative behaviour during treatment [1] and the likelihood of avoiding dental visits in the future [26].

The present study is the first study that aimed to assess the anaesthetic efficiency of ILI and INB using 3% mepivacaine hydrochloride for extraction of mandibular anterior and premolars teeth.

Mepivacaine’s milder vasodilating properties allow for the use of higher concentrations of plain mepivacaine. The 3% mepivacaine provides a shorter onset time for pulpal anaesthesia, and its depth of local anaesthesia is effective in meeting pain control needs, particularly for patients with cardiovascular conditions [6, 27]. 

Given the variable and frequently high failure rates of the inferior alveolar nerve block technique, along with its numerous potential complications [28–30] range from frequent to uncommon complications. Pain and trismus, which can arise from rupturing the mucosa during needle insertion or withdrawal [31, 32], are frequent problems. A less common but noteworthy consequence is injection site needle breakage [33] and facial paralysis, which is usually brought on by the anaesthetic solution getting deposited in the parotid region when the needle is inserted too posteriorly near the mandibular border [34, 35]. Damage to blood vessels or intravascular anaesthetic injections can also result in hematomas. Additional issues that have been documented include diplopia, aphonia, necrosis of the chin skin, ptosis, and abducent nerve palsy [27, 28, 36–38]. Alternative techniques have been explored in the literature to reduce the risk of developing these complications [39–42]. 

Although ILI provides an advantage over regional nerve blocks by inducing anesthesia in only one or two teeth and requires only a small volume of local anesthetic solution so systemic toxicity is reported rarely but on the other hand some patients report tenderness at the injection site for a day or two after treatment [43–46], its efficacy can be unreliable if the needle is not positioned precisely [47], also it is not recommended for ILI into inflamed or infected periodontal sites [48]. There is no specific guidance regarding antibiotic prophylaxis when administering the PDL injection. However, even when it has been administered through healthy periodontal tissue, the PDL injection has induced bacteremia which is mainly in children and that is why children was excluded from our study [49]. 

In this study, pain scores for two techniques were compared during the injection and extraction of mandibular anterior teeth and premolars. The VAS pain score was selected for its ease of use, as well as its superior sensitivity and validity compared to the verbal rating scale (VRS) [50]. Statistical analysis showed that the mean VAS score during ILI was lower than during INB. Likewise, the mean VAS for pain during tooth extraction in the ILI group was lower than in the INB group, despite both techniques being effectively employed for the extraction of mandibular anterior teeth and premolars as an alternative to the inferior alveolar nerve block.

In contrast to an earlier study by the same author, which examined the effects of 2% lignocaine with adrenaline 1:200,000 as an anaesthetic agent in intraligamentary injection (ILT) and mental incisive nerve block injection (MINBT), the current results show differing findings regarding VAS. When assessing pain at the time of injection, the mean VAS score was 2.60 in group MINBT and 2.33 in group ILT with p value = 0.004 which was statistically significant, while the mean score during extraction was found to be higher group ILT (2.47) than for group MINBT (1.47) with p value = 0.039 [51]. This discrepancy may be attributed to the use of 3% mepivacaine as the anaesthetic agent during extraction.

Previous studies have independently investigated the ILI and INB techniques with different assessment methods. Several authors have noted that periodontal injection can achieve sufficient anesthesia and may serve as an alternative to IANB, offering the advantages of a lower anesthetic dose and minimizing soft tissue anesthesia compared to IANB and INB.

Malamad in 1982, evaluated PDL injection for mandibular anesthesia in isolated region by using both a conventional syringe and two devices designed for this procedure. A high success rate was achieved, with a low incidence of adverse reaction and highly favorable comment from both patients and administrators, he recommended further study to determine the response of periodontal and pulpal tissues [52]. Also, a study evaluating the anaesthetic efficacy of 2% lidocaine with different concentrations of epinephrine (1:80,000 and 1:200,000) in intraligamentary injection after a failed primary inferior alveolar nerve block showed the success rate in patients receiving supplementary intraligamentary injections in 1:80,000 epinephrine group was 82%, while the intraligamentary injections with 2% lidocaine with 1:200,000 epinephrine were successful in 57% of cases in patients had failed IANB. The difference was statistically significant (*p* = 0.011) [21].

An observational study of intraligamentary local anaesthesia for posterior mandibular was conducted evaluation using Numeric rating scale (NRS) scores for pain analysis, the results showed that all patients tolerated extractions with ILA. NRS pain scores at the extraction appointment were all less than 3 representing little or no pain. Patients did not record an NRS of 0, whereas 61% (*n* = 155) reported a score of 1 and 39% (*n* = 99) reported a score of 2 [22].

The findings of the current study are consistent with a prior investigation compared the efficacy of intraligamentary injections (ILI) to inferior alveolar nerve blocks (IANB) in dental anaesthesia. The study found a significantly significant difference (*P* < 0.001) between the mean VAS values for the ILI group and the IANB group, which were 1.40 ± 1.10 and 3.60 ± 0.56, respectively. According to the study’s findings, ILI is a less invasive and safer option for mandibular extractions than IANB, particularly for those who have bleeding disorders [53]. 

Another study comparing the onset, success rate, injection pain, and post-injection discomfort of inferior alveolar nerve block (IANB) and mental/incisive nerve block (MINB) found that MINB produced anaesthesia considerably faster and had much less injection pain (*p* < 0.05). Nonetheless, within the first four days following injection, the MINB group experienced much more post-injection discomfort (*p* < 0.001). In spite of this, the average pain ratings fell rapidly over time and stayed in the “mild pain” range, in line with other research findings [15, 54]. 

In 2019, MDAS-DEP was utilized as a validated tool to assess anxiety for patient undergoing tooth extraction [23]. In this current study, the mean MDAS-DEP score in the INB group was slightly higher (15.86 ± 3.156 during injection and 11.26 ± 3.60 during extraction) than the ILI group, which had scores of 15.68 ± 3.40 and 10.94 ± 3.16, respectively, with no significant difference when comparing the two group. These results are consistent with a previous study analyse the amount of anxiety and fear felt before, immediately after, and one week after, dental extraction. The results obtained with (MDAS) immediately after extraction concluded that specific type of anaesthetic injection was found to affect patient anxiety, and patients who required specific block type local anaesthesia reporting significantly higher anxiety immediately after the procedure [55]. 

In contrast, a systematic review was conducted in 2016 found discrepancies regarding the time after the procedure as a predictor of anxiety. While majority of the articles showed single set of findings [56–60] than what offered by López-Jornet et al. [55]. This discrepancy could be caused by the fact that the majority of these research focused on extracting impacted third molars, which might have an impact on the anxiety scale. Thus, more research employing a variety of scales to measure patients’ anxiety levels is necessary.

Both the ILI and INB techniques offer the advantage of delivering effective and deep anaesthesia, and they are generally well-tolerated by patients. However, the ILI technique stands out for being less painful during both the injection and extraction phases. Additionally, unlike the IANB and INB techniques, ILI minimizes soft tissue anaesthesia, making it a more comfortable option for patients during dental procedures. This reduced discomfort and limited impact on surrounding soft tissues can enhance the overall patient experience, potentially improving compliance and reducing anxiety associated with dental treatments.

Although the study has several strengths, including a large sample size and standardized investigative procedures, there are some limitations. Factors such as variations in patients’ satisfaction when answering questions and differences in individual pain thresholds could impact the results. Future studies might consider using a split-mouth technique to standardize patient responses and employing different anaesthetic agents with the same participants. Further research, using different scales to assess patient anxiety level and including molar extractions, would be necessary to make more comprehensive conclusions about the relative effectiveness anaesthesia in reducing patient anxiety level.

## Conclusion

In conclusion, by analysing the results obtained, it can be concluded that ILI provides less painful injections when compared to the INB during extraction of mandibular anterior and premolar teeth. It overcomes the side effects of INB as it eliminates lip biting events as well. Care should be taken when using ILI in cases of infection and inflammation.

## Electronic supplementary material

Below is the link to the electronic supplementary material.

Supplementary Material 1

Supplementary Material 2

## Data Availability

Data is provided within the manuscript or supplementary information files.
