# Supplementary material for: Anaesthetic efficacy of intraligamentary injection compared to incisive nerve block using 3% mepivacaine hydrochloride: a randomized clinical trial
Source: BMC Oral Health. 2025 Jan 17;25:90. doi: 10.1186/s12903-024-05147-z (PMC11740491; doi:10.1186/s12903-024-05147-z)
Supplement: Supplementary file 1 — Supplementary Material 1 [file 12903_2024_5147_MOESM1_ESM.docx]

# **CAN YOU TELL US HOW ANXIOUS YOU GET, IF AT ALL,**

# **WITH YOUR DENTAL VISIT?**

## PLEASE INDICATE BY INSERTING ‘X’ IN THE APPROPRIATE BOX

| 1. **If you were told that one of your teeth had to be extracted, how would you feel??** | | | | | |
| --- | --- | --- | --- | --- | --- |
|  | *Not*  *Anxious* ⬜ | *Slightly*  *Anxious* ⬜ | *Fairly*  *Anxious* ⬜ | *Very*  *Anxious* ⬜ | *Extremely*  *Anxious* ⬜ |
|  | | | | | |
| 1. **If you were about to go to the dentist tomorrow to have your tooth extracted, how would you feel?** | | | | | |
|  | *Not*  *Anxious* ⬜ | *Slightly*  *Anxious* ⬜ | *Fairly*  *Anxious* ⬜ | *Very*  *Anxious* ⬜ | Extremely *Anxious* ⬜ |
|  | | | | | |
| 1. **If you were sitting in the waiting room waiting for your dental extraction procedure, how would you feel?** | | | | | |
|  | *Not*  *Anxious* ⬜ | *Slightly*  *Anxious* ⬜ | *Fairly*  *Anxious* ⬜ | *Very*  *Anxious* ⬜ | Extremely *Anxious* ⬜ |
|  | | | | | |
| 1. **If you were about to get a local anaesthesia injection in the gum, how would you feel?** | | | | | |
|  | *Not*  *Anxious* ⬜ | *Slightly*  *Anxious* ⬜ | *Fairly*  *Anxious* ⬜ | *Very*  *Anxious* ⬜ | Extremely *Anxious* ⬜ |
|  | | | | | |
| 1. **If your third molar was about to be removed through a surgical procedure, how would you feel?** | | | | | |
|  | *Not* Anxious ⬜ | *Slightly* Anxious ⬜ | *Fairly* Anxious ⬜ | *Very* Anxious ⬜ | ExtremelyAnxious ⬜ |

#### _________________________________________________________________

#### Instructions for scoring (remove this section below before copying for use with patients)

*The Modified Dental Anxiety Scale*. Each item scored as follows:

Not anxious = 1

Slightly anxious = 2

Fairly anxious = 3

Very anxious = 4

Extremely anxious = 5

Total score is a sum of all five items, range 5 to 25: Cut off is 19 or above which indicates a highly dentally anxious patient, possibly dentally phobic
